# Enhanced Lycopene Extraction from Tomato Peels by Optimized Mixed-Polarity Solvent Mixtures

**DOI:** 10.3390/molecules25092038

**Published:** 2020-04-27

**Authors:** Antonio Zuorro

**Affiliations:** Department of Chemical Engineering, Materials and Environment, Sapienza University, 00184 Rome, Italy; antonio.zuorro@uniroma1.it

**Keywords:** lycopene, extraction, tomato, antioxidant, solvent, mixture design

## Abstract

Mounting evidence from clinical and epidemiological studies suggests that lycopene, the most abundant carotenoid in tomatoes, may be beneficial in the prevention or treatment of some important diseases. Ripe tomato peels are the richest source of lycopene, but the use of conventional solvent extraction methods without pretreatment of the plant material results in very poor recovery. The reason lies in the localization of lycopene in the plant tissue and the low permeability of the latter to solvent molecules. In this paper, a mixture design procedure was used to formulate solvent mixtures allowing the recovery of lycopene from non-pretreated tomato peels. Two ternary systems were investigated: (a) *n*-hexane–ethanol–acetone and (b) ethyl lactate–ethanol–acetone. Optimization of the ternary mixture composition led to a recovery of over 90% of the lycopene present in the peels. The high extraction efficiency was explained in terms of lycopene affinity combined with the ability to swell the plant material. A tomato oleoresin with high antioxidant activity and a lycopene content of about 13% (*w/w*) was also produced. Overall, the results indicate that highly effective solvents for direct recovery of lycopene from tomato peels can be easily prepared by a mixture design approach.

## 1. Introduction

Over the last decades, lycopene, the carotenoid pigment responsible for the deep red color of tomato fruits, has received increasing attention from the scientific community due to its reported beneficial effects. Results from epidemiological and clinical studies suggest that lycopene may provide protection against cardiovascular disease [1,2], cancer [3,4,5], and neurodegenerative disorders such as Alzheimer’s, Parkinson’s, and Huntington’s diseases [6,7]. The mechanisms by which lycopene exerts its action have not been fully elucidated, but it is generally believed that they are primarily related to its high antioxidant activity [8,9]. In fact, lycopene is a powerful antioxidant and oxidative stress is often associated with the development of the above diseases. Recently, it has been suggested that mechanisms other than antioxidative protection could also be involved. For example, mounting evidence supports the ability of lycopene to induce apoptosis, inhibit cell proliferation, and increase intercellular gap-junctional communication [10,11,12].

**Figure 1:**
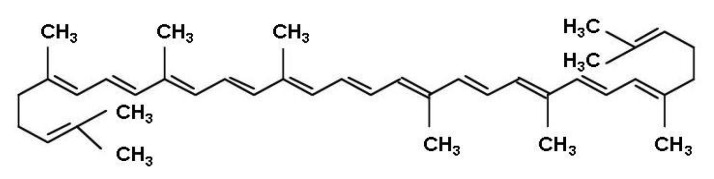
Chemical structure of lycopene (ψ,ψ–carotene).

Chemically, lycopene (ψ,ψ–carotene) is an acyclic tetraterpenic hydrocarbon with 13 carbon–carbon double bonds, 11 of which are conjugated in a linear fashion (Figure 1). This high degree of conjugation imparts strong antioxidant and free-radical quenching properties to the molecule. Lycopene is especially effective in quenching singlet oxygen (^1^O_2_), a highly reactive species capable of damaging various biological components such as lipids, proteins and nucleic acids [13]. Furthermore, it can inhibit free radical damage to LDL cholesterol [14], preventing its oxidation and deposition in the arterial wall, where atherosclerotic plaques form [15].

The above observations have boosted interest in developing supplements, functional foods, and cosmetic products containing lycopene. As a result, the lycopene market has experienced a rapid growth, both in size and value [16]. In parallel, efforts have been made to develop new technologies, or improve those already in use, for the recovery of this carotenoid from natural sources [17], including agroindustrial wastes [18,19,20].

Tomato peels are an abundant byproduct of the tomato processing industry and a very rich source of lycopene. However, the localization of the pigment within the thylakoid membranes of the chloroplasts and the compactness of the tomato peel tissue make it hardly accessible to the extraction solvent. Accordingly, conventional solvent extraction technologies are usually scarcely effective, unless adequate pretreatments, such as those based on ultrasounds [21], microwaves [22] or enzymes [23], are used. These methods cause a partial disintegration of the plant material favoring the release of lycopene into the solvent. Nevertheless, their implementation can have a large impact on the process costs and/or affect the properties of the extracted compound(s).

In this paper, we present a different approach for the recovery of lycopene from tomato peels not subjected to any treatment. It is based on the use of homogeneous solvent mixtures exhibiting two distinct properties: (a) high lycopene affinity and (b) ability to swell the plant material and thus enhance solvent penetration. Since lycopene is a non-polar compound, a good extraction solvent should be non-polar or slightly polar and have a low boiling point to allow easy removal by evaporation. Conversely, a good swelling agent should have a relatively high polarity [24,25]. Therefore, we focused on mixed-polarity solvent mixtures. The first objective of this study was to assess whether the mixture design methodology could be used to formulate an optimal solvent mixture. Second, we were interested in evaluating the characteristics of the solid product obtained by using the optimized mixture as extraction solvent. The results obtained indicate that the mixture design procedure can be conveniently used to formulate solvent mixtures allowing the recovery of lycopene from untreated tomato peels.

## 2. Results and Discussion

### 2.1. Solvent Effects on Lycopene Extraction

During the ripening of tomato fruit, a number of physiological and morphological changes occur, with a progressive disappearance of chlorophyll and the increased synthesis and accumulation of lycopene in the differentiating chloroplasts [26]. In order to prevent the detrimental effects of an excess of the carotenoid on cellular functions, lycopene is sequestered in the form of crystals within the membranes of plastids. This process is mediated by fibrillin, a glycoprotein that is highly expressed in the ripening fruit [27]. In its crystalline state, lycopene is very stable. In addition, due to the low permeability of the peel tissue to solvent molecules, it is hardly accessible to extracting solvents.

In the present study, two ternary solvent systems were investigated: (a) *n*-hexane–ethanol–acetone and (b) ethyl lactate–ethanol–acetone, with the aim to use them for recovering lycopene from non-pretreated tomato peels.

In the first system, *n*-hexane was selected due to its high affinity for lycopene, while ethanol and acetone were included because of their ability to cause swelling of cellulosic materials [24,28]. Acetone and, to a lesser extent, ethanol, have also a certain affinity for lycopene [29], which further supports their presence in the extraction solvent. In this regard, it is noteworthy that one of the most common analytical methods for the determination of lycopene in plant materials utilizes *n*-hexane–ethanol–acetone mixture at 50:25:25 (*v/v*) as extraction solvent [30,31]. However, according to a study performed on whole tomatoes, tomato sauce, and tomato paste, the optimal solvent composition can vary significantly with the type of tomato product used [32].

In the second system, *n*-hexane was replaced by ethyl lactate, an environmentally friendly solvent produced from renewable raw materials and approved by the FDA for food use [33]. Recent studies on tomato processing waste [29] and dried tomato powder [34] showed that ethyl lactate is a good lycopene solvent.

Extraction yields (*y*) were calculated as:

(1) $$y=100\frac{c_{e}\times \;V}{c_{t}\times \;m}$$

where *c_e_* is the concentration of extracted lycopene in the solvent, *V* is the volume of the solvent, *c_t_* is the total lycopene content of the peels and *m* is the dry weight of the peels. The complete experimental design layout for the two ternary systems at 10, 25, and 30 °C is reported in Table 1 and Table 2.

Total lycopene content of tomato peels was 272 ± 28 mg per 100 g of dry material. This value is in line with those determined in previous studies from our laboratory using fresh tomato fruits [35] or tomato processing waste [18,23,36]. Nevertheless, it is important to remember that lycopene content of tomato fruits can be affected by production variables such as tomato cultivar, ripening stage, and cultivation conditions [37,38,39].

The values in Table 1 and Table 2 indicate that temperature had a significant positive effect on lycopene extraction. At each temperature, *n*-hexane–ethanol–acetone mixtures resulted in higher recovery efficiencies, up to about 98%. For the ethyl lactate–ethanol–acetone system, they varied from about 3% to 82%.

The experimental data were analysed by different Scheffé polynomial models (linear, quadratic, cubic, and special cubic). The best result was obtained with the special cubic model:

(2) $$y=\underset{i}{\sum }a_{i}x_{i}+\underset{i}{\sum }\underset{j}{\sum }a_{ij}x_{i}x_{j}+a_{123}x_{1}x_{2}x_{3}$$

where *y* is the lycopene extraction yield and *x_i_* is the weight fraction of the *i*th mixture component (0 ≤ *x_i_* ≤ 1, with the constraint: Σ*_i_ x_i_* = 1). The coefficients *a_i_* reflect the contributions of pure components to the extraction yield, while *a_ij_* and *a_123_* are related to the combined effects of two or three components. Positive or negative values indicate synergistic or antagonistic effects, respectively.

**Figure 2:**
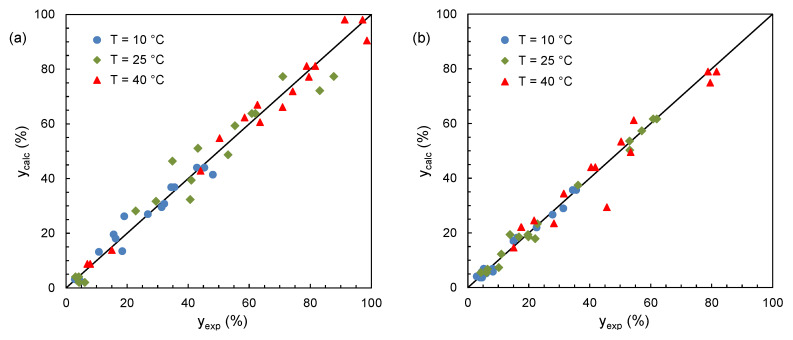
Comparison between experimental (*y_exp_*) and calculated (*y_calc_*) extraction yields for: (**a**) *n*-hexane–ethanol–acetone and (**b**) ethyl acetate–ethanol–acetone.

Equation (2) contains seven unknown parameters, which were estimated by the least squares method (Tables S1 and S2). ANOVA analysis of the results showed that they were statistically significant (*p* < 0.001). Figure 2 reveals the good agreement between experimental and calculated results, the adjusted coefficient of determination (adj-*R^2^*) ranging from 0.907 to 0.977.

**Figure 3:**
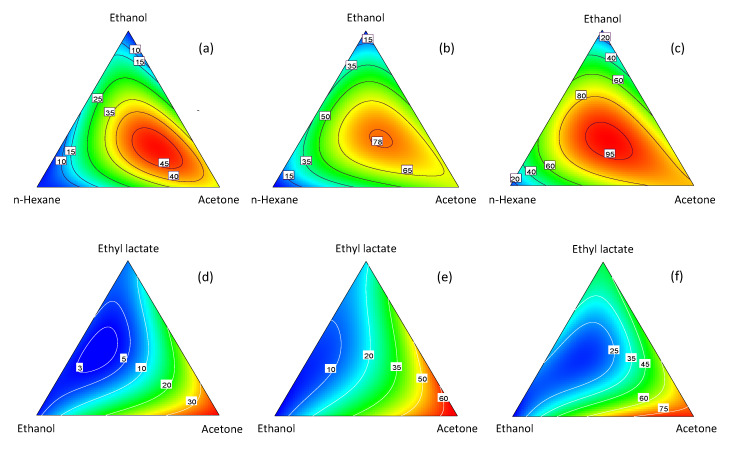
Contour plots for lycopene extraction yields using *n*-hexane–ethanol–acetone at: (**a**) 10 °C, (**b**) 25 °C and (**c**) 45 °C, and ethyl lactate–ethanol–acetone at: (**d**) 10 °C, (**e**) 25 °C, and (**f**) 40 °C.

**Figure 4:**
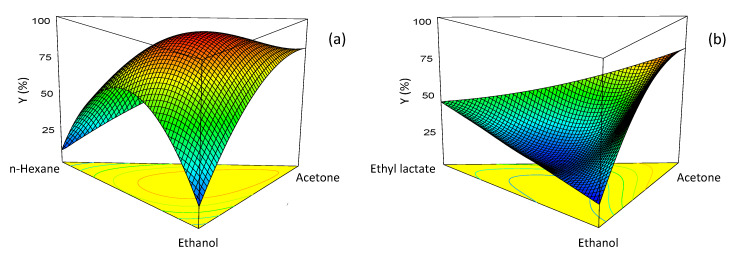
Response surface plots for lycopene extraction yields at 40 °C using: (**a**) *n*-hexane–ethanol–acetone and (**b**) ethyl lactate–ethanol–acetone.

The model equation was used to generate response surface and contour plots. Figure 3 shows the contour plots for the two systems at the three temperatures, while in Figure 4 two representative response surface plots are displayed. An examination of these plots reveals some interesting differences in the behavior of the two systems. For the *n*-hexane–ethanol–acetone system, the extraction yield exhibited a sharp maximum. The position of the maximum was little affected by temperature, while the yield value increased considerably with the temperature. In contrast, in the ethyl lactate–ethanol–acetone system, pure acetone was always the best solvent.

The appearance of a maximum in the model response arises from the positive sign of interaction coefficients and reveals synergism between mixture components. When *n*-hexane was replaced by ethyl lactate, some interaction coefficients became negative, suggesting that antagonistic effects were present.

To estimate the contribution of solvent–lycopene affinity to the carotenoid recovery, the solubility parameters of lycopene and solvents were compared. In fact, it is known that the closer the solubility parameters of two components, the higher their affinity.

According to the classic solubility theory developed by Hildebrand and Scott [40], the solubility parameter of a substance (δ) is given by the square root of the cohesive energy density:

(3) $$\delta =\sqrt{\frac{\Delta E}{v_{m}}}$$

where Δ*E* is the cohesive energy of that substance and *v_m_* its molar volume.

Hansen proposed to break down the overall cohesive energy into three contributions related to the dispersion energy, the polarity energy and the hydrogen-bonding energy [41]. As a result, the total solubility parameter can be expressed as:

(4) $$\delta^{2}=\delta_{D}^{2}+\delta_{P}^{2}+\delta_{H}^{2}$$

where *δ_D_, δ_P_,* and *δ_H_* represent the dispersion, the polar and the hydrogen-bonding solubility parameters. For mixed solvents, the solubility parameter can be determined as follows:

(5) $$\delta_{\mathrm{mix}}=\sum_{i}\varphi_{i}\delta_{i}$$

where *φ_i_* is the volume fraction of the *i*th component in the solvent mixture and *δ_i_* is its solubility parameter.

At this point, the affinity of a solute (A) for a solvent (B) can be quantified by calculating the distance (*D*) in Hansen space between the points representing the solute (*δ_D,A_, δ_P,A,_ δ_H,A_*) and the solvent (*δ_D,B_, δ_P,B,_ δ_H,B_*):

(6) $$D=\sqrt{{\left(\delta_{D,A}-\delta_{D,B}\right)}^{2}+{\left(\delta_{P,A}-\delta_{P,B}\right)}^{2}+{\left(\delta_{H,A}-\delta_{H,B}\right)}^{2}}$$

Small *D*-values indicate that the molecular interactions between A and B are similar, and hence that they exhibit high affinity. The solubility parameters and *D*-values for lycopene and the four solvents are given in Table 3, from which the following order of affinity for lycopene can be established: *n*-hexane > acetone > ethyl lactate > ethanol. This order does not reflect that observed for lycopene recovery. In particular, *n*-hexane, the solvent with the highest affinity for lycopene (*D* = 0.7 MPa^0.5^), resulted in the lowest yields (from about 3%, at 10 °C, to about 7% at 40 °C). In contrast, the most effective solvent mixtures had relatively large *D*-values (Table 4). Thus, it can be inferred that the extraction process was also influenced by factors other than solute–solvent affinity.

An important aspect that deserves attention is the effect of solvent components on the plant matrix. In cellulosic materials, cellulose is organized in microfibrils containing both crystalline and amorphous regions. Microfibrils are assembled into fibers of larger diameter that are cross-linked by hemicelluloses and embedded in a gel-like pectic matrix [42]. The degree of cellulose crystallinity and the spatial organization of the cellulose/hemicellulose network are mainly determined by intra- and intermolecular hydrogen bonds. These bonds are formed between hydroxyl groups present in the β-1,4-linked d-glucopyranose units of cellulose [43]. Solvent molecules of small size and high polarity can penetrate the plant matrix and adsorb on these hydroxyl groups. Following adsorption, some bonds are broken, increasing the distance between the cellulose fibers and causing the material to swell [44]. In most cases, swelling is limited to the amorphous regions of cellulose, which are more reactive and accessible to solvent.

The detailed mechanisms of swelling are currently not fully understood, but the evidence so far indicates that this phenomenon is mainly affected by three solvent properties: hydrogen bonding ability, basicity and molar volume [44,45]. The protic or aprotic nature of the solvent also seems to play an important role in the swelling process [24]. This is because protic solvents, such as water and ethanol, can act as hydrogen bond donors and acceptors, while the aprotic ones, such as acetone and dimethyl sulfoxide, can only act as hydrogen bond acceptors.

Going back to the systems investigated, it can be noted that they share two of the three components, namely, acetone and ethanol. Both, especially ethanol, are good swelling agents [44,46]. The higher swelling ability of ethanol can be explained by its lower molar volume (58.5 against 74 cm^3^/mol) and its higher hydrogen bonding capacity (see Table 3). This makes ethanol more effective in penetrating the cellulosic material and breaking intermolecular bonds. Concerning the molecular size, solvents with a molar volume >100 cm^3^/mol are very poor swelling agents, even when capable of strong hydrogen bonding [45]. In mixed solvents, the swelling behavior is much more complex, as solvent components can interact with each other and/or cause solvent restructuring.

The above considerations suggest a possible explanation of the results. For the *n*-hexane–ethanol–acetone system, it can be assumed that the presence of ethanol and acetone allows the tomato tissue to swell, favoring the penetration of *n*-hexane into the matrix and the solubilization of lycopene. Due to the partial affinity of acetone and ethanol for lycopene, also these components can contribute to its recovery. Compared to ethanol, acetone has higher affinity for lycopene, but is a less effective swelling agent [46]. Studies performed on the ethanol–acetone system using ^1^H NMR spectroscopy and ab initio calculations have shown that the molecular solution behavior of this system is determined by different types of intermolecular interactions [47,48]. They include hydrogen bonding between ethanol or acetone molecules, hydrogen bonding between ethanol and acetone molecules and dipole–dipole interactions between acetone molecules. From the association of ethanol molecules, dimers, and cyclic multimers (especially trimers and tetramers) can be formed [49]. At concentrations below 55 mol%, acetone tends to self-associate rather than form hydrogen bonds with ethanol, while self-association of ethanol molecules is more favorable at ethanol concentrations below 50 mol% [47]. The distribution of free and bound solvent molecules can influence the kinetics and the extent of the extraction process, as only free ethanol or acetone molecules can induce swelling and/or reach lycopene. Therefore, the maximum in the dependence of the extraction yield on solvent composition for the *n*-hexane–ethanol–acetone system could result from an optimal ethanol/acetone ratio allowing maximum swelling and access of *n*-hexane to lycopene.

The molecular behavior of the ethyl lactate–ethanol–acetone system is fundamentally different. As already mentioned, ethyl lactate is a good solvent for lycopene but a quite poor swelling agent [45]. Thus, the use of ethyl lactate as single solvent cannot lead to high recovery of lycopene from tomato peels. Addition of increasing amounts of ethanol caused a decrease in extraction efficiency, while the opposite was true for acetone. This can be explained by the fact that the ethyl lactate molecule contains one hydrogen bond donor site and three hydrogen bond acceptor sites [50], which makes it capable of forming several intra- and intermolecular hydrogen bonds [51,52]. As reported by Qiao et al. [53], complexes of ethyl lactate with acetone and ethanol can be formed [53]. These complexes can significantly reduce the fraction of free ethanol and acetone molecules, with a decrease in the swelling capacity of the solvent and the number of ethyl acetate molecules capable of reaching lycopene.

To sum up, the following conclusions can be drawn: (a) the existence of an optimal mixture composition in the *n*-hexane–ethanol–acetone system is a result of the high affinity of *n*-hexane (and, to a lesser degree, acetone) for lycopene, combined with the swelling ability of acetone and ethanol; (b) in the ethyl lactate–ethanol–acetone system, the formation of complexes of ethyl lactate with acetone and ethanol can limit the extent of swelling and the penetration of ethyl lactate into the plant tissue, leading to a lycopene recovery lower than that with pure acetone.

### 2.2. Optimization of Mixture Composition

Optimization of the *n*-hexane–ethanol–acetone mixture composition was performed numerically, giving the results summarized in Table 5. The optimal extraction yield increased with temperature from 45.18%, at 10 °C, to 98.56%, at 40 °C. In contrast, the composition of the optimized mixture was not appreciably affected by temperature.

To validate the model predictions, additional experiments were performed with the optimized mixtures. The average prediction error was around 2% (Table 5). Since the highest extraction yield was achieved at 40 °C with a mixture consisting of 30.6% (*w/w*) *n*-hexane, 32.8% ethanol and 36.6% acetone, this mixture was used to produce the tomato oleoresin in the mechanically stirred extractor.

### 2.3. Production of Tomato Oleoresin

Preliminary tests were carried out in the 1.5-L stirred extractor to evaluate the influence of liquid-to-solid ratio (LSR) on lycopene recovery. In these experiments, tomato peels were contacted at 40 °C for 30 min with the optimized mixture. LSR was varied between 5 and 30 mL/g, leading to the results shown in Figure S1. As can be seen, an increase in LSR improved the lycopene extraction yield, but above 20 mL/g no appreciable changes in yields were observed. Accordingly, the production of tomato oleoresin was carried out at LSR = 20 mL/g.

Under optimal conditions, the amount of oleoresin produced was 26.8 ± 1.5 g per kg of dry tomato peels. The lycopene content of the oleoresin was 12.7 ± 1.2 (wt%) and its antioxidant capacity was 1582 ± 49 μmol TE/g. Furthermore, about 91.5% of the lycopene present in the peels was recovered. This value is lower than that determined in stirred flasks (*y* = 96.52%), which could be due to less efficient mixing in the extractor and/or to possible lycopene losses during solvent removal and evaporation. However, the lycopene titer of the oleoresin is considerably high, making it a product of interest for a variety of applications.

## 3. Materials and Methods

### 3.1. Chemicals and Plant Material

Acetone (CAS No. 67-64-1), methanol (CAS No. 67-56-1), ethanol (CAS 64-17-5), ethyl acetate (CAS No. 141-78-6), ethyl lactate (CAS No. 687-47-8) and *n*-hexane (CAS No. 110-54-3) were obtained from Carlo Erba (Milano, Italy). Butylated hydroxytoluene (BHT, CAS No. 128-37-0), potassium persulfate (CAS No. 7727-21-1), Trolox [6-hydroxy-2,5,7,8-tetramethylchroman-2-carboxylic acid] (CAS No. 53188-07-1) and ABTS [2,2’-azino-bis(3-ethylbenzothiazoline-6-sulphonic acid) diammonium salt] (CAS No. 30931-67-0) were provided by Sigma–Aldrich (St. Louis, Mo, USA). All chemicals were of analytical grade and used as received.

Fresh ripe tomatoes of the variety *Piccadilly* were purchased from a local market and stored in the dark at 4 °C. Before each set of experiments, a few tomato fruits were immersed in boiling water for 1–2 min, cooled in tap water and hand peeled. Tomato peels were left to dry in air for about 2 h and then characterized for total lycopene and moisture content.

Natural lycopene standard (10% by weight) was obtained from LycoRed Natural Products Industries Ltd. (Beer-Sheva, Israel).

### 3.2. Analytical Methods

Lycopene concentration in the solvent was determined using a UV 2700 spectrophotometer (Shimadzu, Kyoto, Japan). In the visible region, the absorption spectrum of lycopene displays three characteristic peaks (Figure S2). To avoid spectral interferences from other carotenoids, optical measurements were made at 503 nm [54].

Total lycopene content was determined according to the procedure of Fish et al. [30] with some modifications [35]. Lycopene extraction was carried out in three consecutive stages using 1 g of tomato peel and a solvent-to-peel ratio in the first, second and third stage equal to 100, 50 and 10 mL g^–1^. The amount of lycopene in the starting material was calculated as the sum of the values obtained in each extraction stage and the total lycopene content was expressed as mg of lycopene per g of dry material.

Antioxidant activity was evaluated by the ABTS method as described by Conde et al. [55] with slight modifications. Briefly, an ABTS^•+^ stock solution was first prepared by reacting 7 mM of the cation radical solution with 2.45 mM potassium persulfate. The reaction mixture was left in the dark at room temperature for 12 h. An aliquot of stock solution was diluted in methanol to achieve an absorbance value of about 0.7 ± 0.02 at 734 nm. A known amount of tomato oleoresin was then dissolved in ethyl acetate, following the procedure reported elsewhere [56]. 100 μL of the resulting solution were added to 3.9 mL of the diluted ABTS^•+^ solution and gently mixed. After 10 min, the absorbance at 734 nm was measured and the antioxidant capacity (*AC*) was calculated as:

(7) $$AC\left(\%\right)=100\frac{A_{c}-A_{S}}{A_{C}}$$

where *A_S_* is the absorbance of the sample and *A_C_* is the absorbance of the control. 

Results were expressed as μmol Trolox Equivalent Antioxidant Capacity (TEAC) per gram of dry sample using a calibration curve obtained with Trolox standards (0.05–50 μmol L^–1^).

Moisture content was determined by oven drying at 105 °C to constant weight.

### 3.3. Lycopene Extraction Experiments

Lycopene extraction experiments were carried out in 50-mL screw-capped flasks immersed in a water bath maintained at the desired temperature (10, 25 or 40 °C). One gram of partially dehydrated tomato peels and 30 mL of the solvent were placed into the flasks and magnetically stirred at 350 rpm for 30 min. After this time, a 2-mL liquid sample was taken, passed through a 0.45-μm nylon filter and analysed for lycopene content.

### 3.4. Oleoresin Production

Tomato oleoresin was produced in an apparatus consisting of a cylindrical 1.5-L jacketed extractor equipped with a mechanical stirrer [36]. The extractor was loaded with 50 g of partially dehydrated tomato peels and 1 L of the solvent. The system was thermostated at 40 ± 0.1 °C and kept under stirring at 500 rpm for 30 min. Then, the organic solvent was recovered and evaporated in a Rotavapor (R-215, BÜCHI Labortechnik AG, Switzerland). Solvent evaporation was carried at 40 °C and P < 20 mbar, with a rotation speed of 100 rpm. The remaining residue (i.e., the tomato oleoresin) was weighed and characterized for lycopene content and antioxidant activity.

### 3.5. Mixture Design

**Figure 5:**
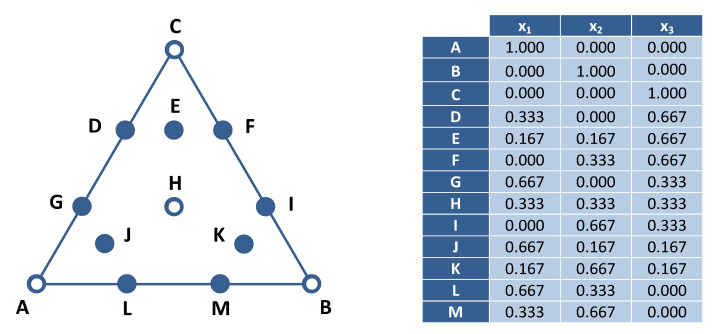
Simplex centroid design for the study of ternary solvent mixtures. Open circles denote duplicates; *x_i_* is the volume fraction of the *i*-th mixture component.

An augmented simplex centroid design (ASCD) was used to investigate the effects of the composition of solvent mixtures on lycopene recovery. The ASCD consisted of a {3,3} simplex lattice, with nine equispaced points on the perimeter of the triangle and three points in the internal region (Figure 5). Experiments at the vertices and at the center point were replicated for the estimation of pure error, leading to a total of 17 runs. They were carried out in random order.

The lycopene extraction yield, expressed as the percentage amount of extracted lycopene relative to the total amount of lycopene in the peels, was taken as the response variable.

The Design-Expert® software (version 7.0, Stat-Ease Inc., Minneapolis, MN, USA) was used to design the experiments and analyse the results.

## 4. Conclusions

The results of this study indicate that the mixture design methodology is a powerful means to formulate solvent mixtures for the efficient recovery of lycopene from untreated tomato peels. The development of an optimized *n*-hexane–ethanol–acetone mixture resulted in a lycopene extraction yield higher than 95%. This mixture also allowed the production of a tomato oleoresin with high lycopene content (12.7 wt%) and antioxidant capacity (1582 μmol TE/g). Another important feature of the proposed methodology is the possibility to detect synergistic or antagonistic effects between mixture components.

Future studies should be directed at providing further insight into the molecular behavior of mixed solvents and their effects on the extraction process. Optimizing the recovery of bioactive compounds from agro-industrial wastes is an important step towards the reduction of their environmental impact and the sustainable transformation of a waste into a resource [57,58,59]. Furthermore, an extraction process that avoids the need for a pre-treatment of the plant material can be expected to preserve the biological activity of the extracted compounds to a greater extent compared to cases where physical or chemical pretreatments are used.

## Figures and Tables

**Table 1:** Experimental design layout and lycopene extraction yields (*y*) at 10, 25, and 40 °C for the ternary system *n*-hexane(1)–ethanol(2)–acetone(3). SO is the standard order of experiments and *x_i_* is the weight fraction of the *i*th component in the solvent mixture.

SO	*x*_1_	*x*_2_	*x*_3_	*y* (%)		
				10 °C	25 °C	40 °C
1	1.000	0.000	0.000	3.09	4.15	6.97
2	0.667	0.333	0.000	18.41	40.69	63.57
3	0.667	0.000	0.333	15.59	41.04	44.08
4	0.333	0.667	0.000	10.79	29.46	58.49
5	0.333	0.333	0.333	42.87	87.71	97.14
6	0.333	0.000	0.667	32.18	55.32	62.72
7	0.000	1.000	0.000	4.69	4.28	15.04
8	0.000	0.667	0.333	16.26	22.82	50.32
9	0.000	0.333	0.667	31.34	53.08	79.55
10	0.000	0.000	1.000	35.55	60.84	78.81
11	0.667	0.167	0.167	26.82	43.19	70.92
12	0.167	0.667	0.167	19.07	34.83	74.25
13	0.167	0.167	0.667	48.09	83.13	98.55
14	1.000	0.000	0.000	2.91	3.10	7.93
15	0.000	1.000	0.000	4.07	6.17	15.04
16	0.000	0.000	1.000	34.45	62.00	81.65
17	0.333	0.333	0.333	45.31	71.01	91.32

**Table 2:** Experimental design layout and lycopene extraction yields (*y*) at 10, 25 and 40 °C for the ternary system ethyl lactate(1)–ethanol(2)–acetone(3). SO is the standard order of experiments and *x_i_* is weight fraction of the *i*th component in the mixture.

SO	*x*_1_	*x*_2_	*x*_3_	*y* (%)		
				10 °C	25 °C	40 °C
1	1.000	0.000	0.000	8.14	16.78	40.46
2	0.667	0.333	0.000	5.99	10.99	31.50
3	0.667	0.000	0.333	14.96	36.19	53.46
4	0.333	0.667	0.000	2.93	6.50	21.74
5	0.333	0.333	0.333	5.92	13.83	17.43
6	0.333	0.000	0.667	27.83	57.09	54.46
7	0.000	1.000	0.000	4.69	4.28	15.04
8	0.000	0.667	0.333	16.26	22.82	50.32
9	0.000	0.333	0.667	31.34	53.08	79.55
10	0.000	0.000	1.000	35.55	60.84	78.81
11	0.667	0.167	0.167	8.22	22.14	45.62
12	0.167	0.667	0.167	4.39	10.17	28.24
13	0.167	0.167	0.667	22.55	53.13	50.30
14	1.000	0.000	0.000	5.20	19.88	41.84
15	0.000	1.000	0.000	4.07	6.17	15.04
16	0.000	0.000	1.000	34.45	62.00	81.65
17	0.333	0.333	0.333	4.61	19.73	17.59

**Table 3:** Molecular properties and Hansen solubility parameters of solvent components, water, and lycopene (MW: molecular weight; *v_m_*: molar volume; δ_i_: Hansen solubility parameters; D: distance between points in Hansen space).

Compound	MW (Da)	*v_m_* (cm^3^/mol)	δ_D_ (MPa^0.5^)	δ_P_ (MPa^0.5^)	δ_H_ (MPa^0.5^)	δ (MPa^0.5^)	D (MPa^0.5^)
Acetone	58.08	74.0	15.5	10.4	7.0	19.9	12.5
Ethanol	46.07	58.5	15.8	8.8	19.4	26.5	21.3
Ethyl lactate	118.13	115.0	16.0	7.6	12.5	21.7	14.6
*n*-Hexane	86.18	131.6	14.9	0.0	0.0	14.9	0.7
Lycopene	536.87	604.2	15.6	0.0	0.0	15.6	–

**Table 4:** Hansen solubility parameters and extraction efficiency of some solvent mixtures (*x_i_*: weight fraction of the *i*th component in the mixture; *y*: extraction yield; δ_mixi_: Hansen solubility parameter of the mixture; D: distance between points in Hansen space).

Mixture	*x*_1_	*x*_2_	*x*_3_	*y* (%)	δ_mix_ (MPa^0.5^)	D (MPa^0.5^)
*n*-Hexane–ethanol–acetone	0.167	0.167	0.667	98.55	19.3	11.5
	0.333	0.333	0.333	94.23	18.7	10.8
Ethyl lactate–ethanol–acetone	0.000	0.000	1.000	80.23	19.9	12.5
	0.000	0.333	0.667	79.55	21.6	14.9

**Table 5:** Optimization of mixture composition for the ternary system *n*-hexane(1)–ethanol(2)–acetone(3). *x_i_* is the weight fraction of the *i*th in the mixture, *y_mod_* is the predicted lycopene extraction yield, *y_exp_* is the measured extraction yield and ε is the percent prediction error.

T (°C)	*x*_1_	*x*_2_	*x*_3_	*y_mod_ (%)*	*y_exp_ (%)*	*ε (%)*
10	0.287	0.277	0.436	45.18	43.85	2.94
25	0.310	0.265	0.425	78.91	79.64	–0.93
40	0.306	0.328	0.366	98.56	96.52	2.07

## Supplementary Materials

The following are available online, Figure S1: Effect of liquid-to-solid ration on lycopene extraction yield, Figure S2: Absorption spectrum of lycopene in the visible region, Table S1: Estimated model coefficients at 10, 25 and 40 °C for the ternary system n-hexane(1)–ethanol(2)–acetone(3), Table S2: Estimated model coefficients at 10, 25 and 40 °C for the ternary system ethyl lactate(1)–ethanol(2)–acetone(3).
