# Supplementary material for: Enhanced Lycopene Extraction from Tomato Peels by Optimized Mixed-Polarity Solvent Mixtures
Source: Molecules. 2020 Apr 27;25(9):2038. doi: 10.3390/molecules25092038 (PMC7248986; doi:10.3390/molecules25092038)
Supplement: Supplementary file 1 [file molecules-25-02038-s001.pdf]

Enhanced Lycopene Extraction from Tomato Peels by Optimized Mixed-Polarity Solvent Mixtures

Antonio Zuorro

Supplementary Material

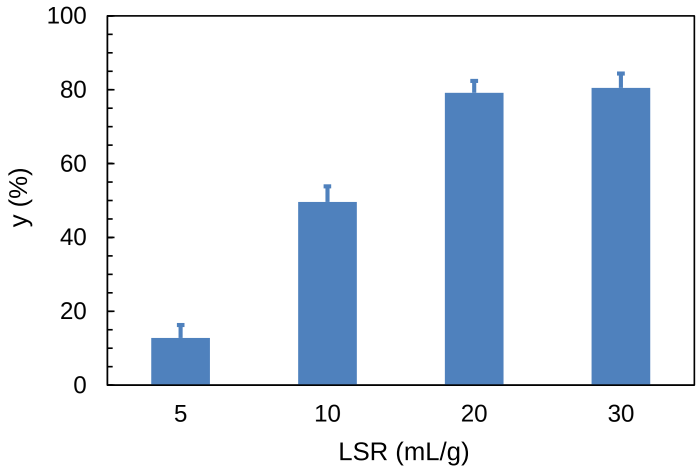

Figure S1. Effect of liquid-to-solid ration (LSR) on lycopene extraction yield (y).

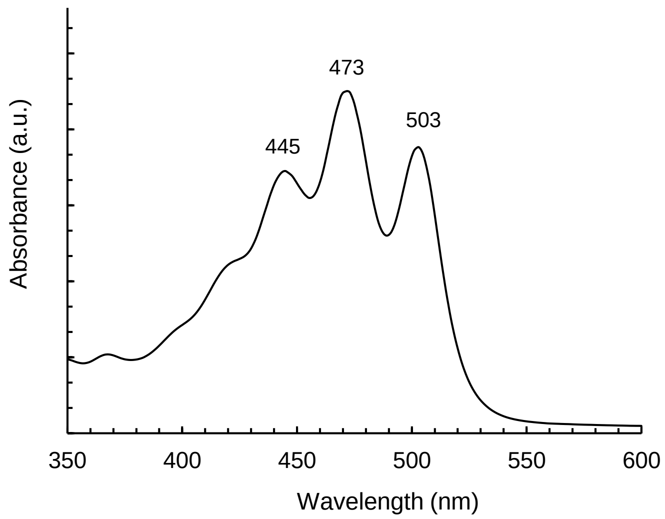

Figure S2. Absorption spectrum of lycopene in the visible region, with its three characteristic peaks.

Table S1. Estimated model coefficients at 10, 25 and 40 °C for the ternary system *n*-hexane(1)–ethanol(2)–acetone(3). SE is the standard error and VIF the variance inflation factor associated at each model coefficient.

	T = 10 °C			T = 25 °C			T = 40 °C		
	**Value**	**SE**	**VIF**	**Value**	**SE**	**VIF**	**Value**	**SE**	**VIF**
a_1	3.16	2.79	1.77	4.01	5.43	1.77	8.74	3.19	1.77
a_2	2.37	2.79	1.77	2.00	5.43	1.77	13.81	3.19	1.77
a_3	36.81	2.79	1.77	63.74	5.43	1.77	81.13	3.19	1.77
a_{12}	47.29	15.81	2.21	130.33	30.77	2.21	225.67	18.11	2.21
a_{13}	23.23	15.81	2.21	69.39	30.77	2.21	44.69	18.11	2.21
a_{23}	18.74	15.81	2.21	24.79	30.77	2.21	83.17	18.11	2.21
a_{123}	537.79	102.77	2.36	785.12	200.05	2.36	654.00	117.74	2.36

Table S2. Estimated model coefficients at 10, 25 and 40 °C for the ternary system ethyl lactate(1)–ethanol(2)–acetone(3). SE is the standard error and VIF the variance inflation factor associated at each model coefficient.

	T = 10 °C			T = 25 °C			T = 40 °C		
	**Value**	**SE**	**VIF**	**Value**	**SE**	**VIF**	**Value**	**SE**	**VIF**
a_1	6.88	1.24	1.77	18.51	2.33	1.91	44.01	4.66	1.77
a_2	3.62	1.24	1.77	5.45	2.33	1.91	14.58	4.66	1.77
a_3	35.67	1.24	1.77	61.64	2.33	1.91	78.96	4.66	1.77
a_{12}	–2.96	7.04	2.21	–11.35	12.71	2.21	0.65	26.40	2.21
a_{13}	2.54	7.04	2.21	32.76	12.71	2.21	–27.73	26.40	2.21
a_{23}	17.76	7.04	2.21	21.88	12.71	2.21	78.18	26.40	2.21
a_{123}	–312.03	45.76	2.36	–379.22	82.63	2.36	–796.43	171.65	2.36
